# Automated measurement of penile curvature using deep learning-based novel quantification method

**DOI:** 10.3389/fped.2023.1149318

**Published:** 2023-04-17

**Authors:** Sriman Bidhan Baray, Mohamed Abdelmoniem, Sakib Mahmud, Saidul Kabir, Md. Ahasan Atick Faisal, Muhammad E. H. Chowdhury, Tariq O. Abbas

**Affiliations:** ^1^ Department of Electrical and Electronic Engineering, University of Dhaka, Dhaka, Bangladesh; ^2^ Department of Electrical Engineering, College of Engineering, Qatar University, Doha, Qatar; ^3^ Department of Surgery, Weill Cornell Medicine-Qatar, Ar-Rayyan, Qatar; ^4^ Urology Division, Surgery Department, Sidra Medicine, Doha, Qatar; ^5^ College of Medicine, Qatar University, Doha, Qatar

**Keywords:** penile curvature, artificial intelligence, machine learning, YOLO, UNET, HRNet, hypospadias, chordee

## Abstract

**Objective:**

Develop a reliable, automated deep learning-based method for accurate measurement of penile curvature (PC) using 2-dimensional images.

**Materials and methods:**

A set of nine 3D-printed models was used to generate a batch of 913 images of penile curvature (PC) with varying configurations (curvature range 18° to 86°). The penile region was initially localized and cropped using a YOLOv5 model, after which the shaft area was extracted using a UNet-based segmentation model. The penile shaft was then divided into three distinct predefined regions: the distal zone, curvature zone, and proximal zone. To measure PC, we identified four distinct locations on the shaft that reflected the mid-axes of proximal and distal segments, then trained an HRNet model to predict these landmarks and calculate curvature angle in both the 3D-printed models and masked segmented images derived from these. Finally, the optimized HRNet model was applied to quantify PC in medical images of real human patients and the accuracy of this novel method was determined.

**Results:**

We obtained a mean absolute error (MAE) of angle measurement <5° for both penile model images and their derivative masks. For real patient images, AI prediction varied between 1.7° (for cases of ∼30° PC) and approximately 6° (for cases of 70° PC) compared with assessment by a clinical expert.

**Discussion:**

This study demonstrates a novel approach to the automated, accurate measurement of PC that could significantly improve patient assessment by surgeons and hypospadiology researchers. This method may overcome current limitations encountered when applying conventional methods of measuring arc-type PC.

## 1. Introduction

Congenital penile curvature (PC) is typically caused by abnormalities in genital development, such as chordee or hypospadias. Approximately 1 in 300 newborn males exhibit hypospadias (1, 2), with an estimated one-third of individuals also presenting with notable PC (3, 4). This condition is thought to result from arrested embryological development of the ventral axis of the penile shaft, often leading to insufficient skin, abnormally short urethral plate, and ventro-dorsal corporeal disproportion (5–7). In some cases, congenital PC may coexist with a normal meatus but deficient urethra, termed chordee without hypospadias (8). Penile curvature can also occur even when the urethra is completely normal, which is thought to affect ∼0.6% of newborn boys (9).

PC may develop in a variety of contexts, although it is more prevalent and appears earlier in patients with hypospadias, necessitating early examination and treatment. In situations of Hypospadias, tiny differences in the degree of PC can significantly impact surgical decision-making and the ultimate choice of repair procedure (10, 11). A prior study of pediatric urologists found that a highly variable fraction chose no intervention when the amount of PC varied from 10° (69%), to 20° (64%), or 30° (16%) (12). At the same time, 66% of the urologists used dorsal correction with a PC of 40°, compared to 47% of respondents for a PC of 50° (13). Notably, around 37% of readings acquired using a goniometer and eye assessment alone may result in needless surgical treatments (14). If not treated properly, PC can persist into adulthood and cause further complex patient issues (11, 15, 16). In order to adequately identify the severity of hypospadias, it is of the utmost essential to assess the degree of PC accurately.

Although PC extent has substantial clinical relevance and predictive significance, evaluation of this disorder is inconsistent across surgeons, with no rapid and reliable measurement techniques available at present (11, 17, 18). Current approaches typically involve visual assessment upon artificial erection induced by saline injection (19). However, recent developments in artificial intelligence (AI) have revolutionized many medical sectors including radiology, pathology, ophthalmology, and cardiology (20–24). Numerous urology subspecialties including endourology, reproductive medicine, stones, hydronephrosis, malignancies, and pediatric urology have already benefited from the use of AI applications, which can be used to perform automatic segmentation, classification, registration, and analysis of medical images (25–27). In this way, AI can provide highly accurate predictions that inform rapid patient diagnosis and treatment decisions. AI tools can outperform conventional statistical methods in terms of prediction accuracy, and if integrated into relevant guidelines, may completely transform the way that urologists make clinical decisions (28, 29).

To measure PC, current methods involve unassisted visual inspection, a goniometer, or mobile app-based angle measurements. However, due to their high subjectivity and poor inter- and intra-observer agreement, all of these procedures are intrinsically flawed (14, 17, 30, 31). During surgery, normal saline is often injected into the penis to assess curvature, which must be quantified in real-time to reduce surgery duration and minimize fluid leakage from the operation site. Considering these major limitations of PC measurement, Abbas et al*.* (32), proposed an automatic quantification method which involved penile area localization, shaft segmentation, and angle calculation using a novel AI-based tool. While localization and segmentation aspects achieved satisfactory results, angle calculation sometimes failed when applied to non-uniform masks. To overcome this limitation, here we developed a novel approach to calculate the axes of the penile shaft using two pairs of key points that no longer depend on arc area. Additionally, to better automate angle measurement, we trained and validated an HRNet-based deep learning model which can measure curvature angles more precisely, even when applied to non-uniform real-life anatomy.

## 2. Method

**Figure 1:**
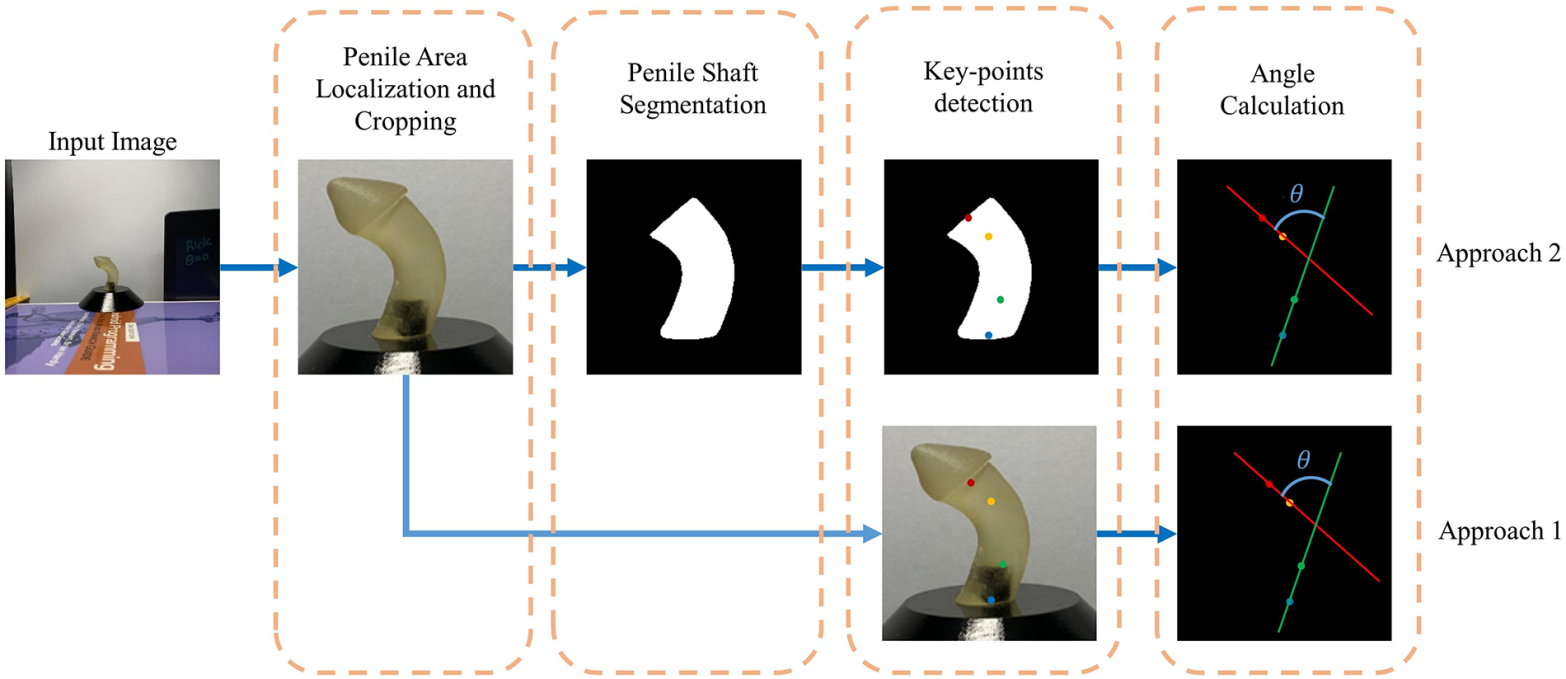
Penile angle measurement pipeline.

Our previous pipeline for autonomous measurement of penile curvature (PC) consisted of three distinct steps: automated localization of the penile area, segmentation of the penile shaft, and angle computation. Due to inadequate performance with real-world cases, in the current study, we developed an alternative pipeline in which we incorporate the earlier steps but focused on detecting key points (as shown in Figure 1). For automated localization of the penile area, a YoloV5l network was trained to predict a bounding box around the relevant region and then crop this to a predefined shape. For the segmentation of the penile shaft, several UNet models (encoder-decoder) including state-of-the-art convolutional neural network (CNN) models were used to create binary masks identifying the penile shaft. For the key point assignment task, a Deep Learning model, HRNet was trained and validated to recognize two pairs of crucial points on the penile shaft (either from cropped pictures or derivative masks). Finally, the curvature angle was computed using two vectors drawn through the vertices of these key points. This end-to-end pipeline automates the whole process of PC measurement which takes the 2D penile model images as input and gives the calculated angle as output. Behind the scene, the trained YOLOv5l model identifies the penile area, the segmentation model generates the penile shaft mask, the HRNet model predicts the vectors through the proximal and distal area of the shaft and the angle between the vectors is calculated automatically to show the penile curvature angle as an output.

### 2.1. Dataset description

The dataset employed in this study was previously described by Abbas et al*.* (32), and consisted of 913 total images generated using *n* = 9 3D-printed penile models with different curvature angles (ranging from 18° to 86°) as shown in Supplementary Figure 1. The models were designed by a 3D model developer before resizing the stereolithography (STL) files to dimensions appropriate for children (1.5 cm wide and 5–6 cm long). The penile models were then photographed with a triple-lens iPhone 11 Pro Max mobile camera with a 12-megapixel resolution. The camera was set 20–25 cm away from each model and moved along the horizontal and vertical axes (−5°, 5°) and (0°, 20°), respectively. For each model around 100 pictures were captured at different camera positions (penile models' angles and number of images are listed in Supplementary Table 1).

### 2.2. Penile area localization

**Figure 2:**
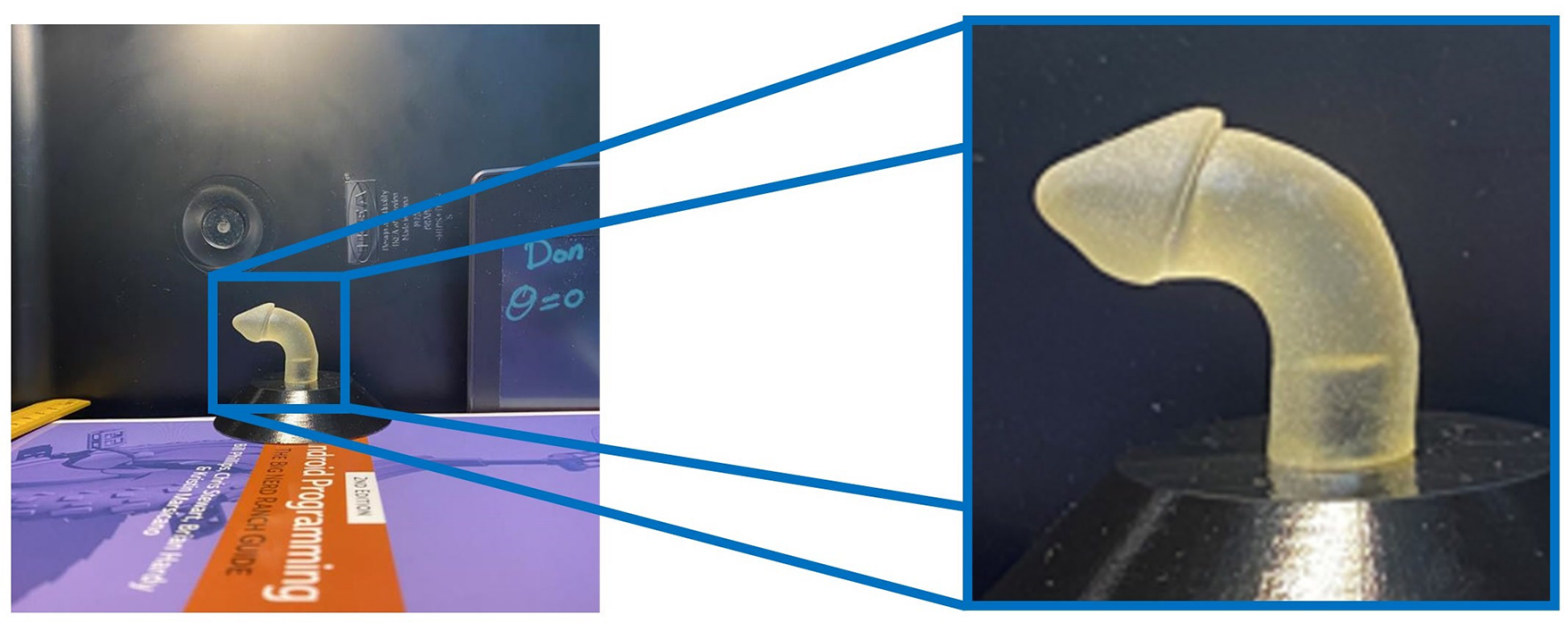
Penile area localization and cropping.

To reduce image complexity, the penile area was localized in each photograph and images were then cropped to retain only this area, thereby eliminating the irrelevant background. Localizing and cropping the penile area also reduced the amount of input data that required processing in subsequent steps of the pipeline, thus making the procedure faster and more efficient (an overview of this process is shown in Figure 2).

We first annotated all 913 images with appropriate bounding boxes and then automated this process using a YOLOv5 model (a single-stage object detector consisting of three components: a Backbone, a Neck, and a Head for making dense predictions). The YOLO (You Only Look Once) technique for identifying objects involves first splitting the picture into a grid of cells and then calculating the probability that an item is located in each of those cells. For each cell that could hold an object, YOLO calculates an estimated bounding box and class. The probability of an object's presence in a given cell is predicted using a Deep Neural Network. Once complete, the model was able to process any raw photograph into an image shaped 256 × 256 pixels consisting of only the penile area.

**Figure 3:**
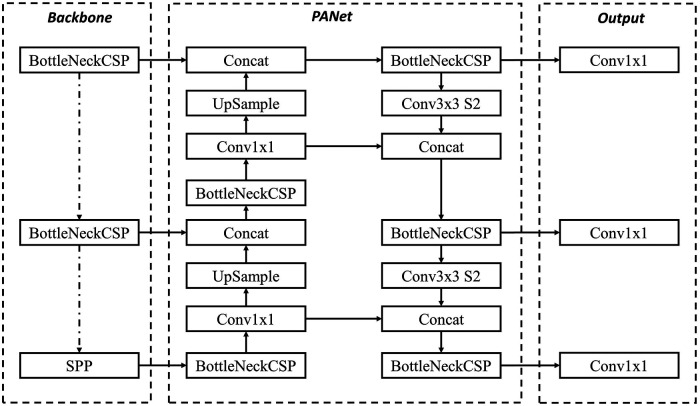
YOLOv5 architecture used for penile area cropping from still 2D images.

All YOLOv5 models are composed of the same 3 components: CSP-Darknet53 as a backbone, SPP and PANet in the model neck, and the head used in YOLOv4 (33). There is no difference between the five YOLOv5 models—nano (n), small (s), medium (m), large (l), and extra-large (x) in terms of operations used (only the number of layers varies). YOLOv5 employs SiLU (Sigmoid Linear Unit) and Sigmoid activation functions. Three outputs are provided by YOLOv5: the classes of the identified objects, their bounding boxes, and objectness ratings (the model's confidence that a particular region in an image contains an object). The class loss and the objectness loss are then computed using BCE (Binary Cross Entropy). CIoU (Complete Intersection over Union) is an improved penalty function, which helps to improve localization accuracy. Additionally, YOLOv5 employs the Focus Layer to replace the first three layers of the network, thereby reducing the number of parameters, floating point operations per second (FLOPS), and Compute Unified Device Architecture (CUDA) memory required. YOLOv5 also eliminates Grid Sensitivity by using a centre point offset range from −0.5 to 1.5 (instead of just 0 to 1) thus allowing the detection of objects in the corners of images. YOLOv5 is written on Pytorch rather than C, giving more flexibility to control encoding operations. The overall architecture of YOLOv5 is shown in Figure 3.

The YOLOv5 architecture is independent of the inference size, safe for stride multiple constraints. Two variables namely *model depth multiple* and *layer channel multiple* are used for model scaling, and compound scaling when used jointly. The depth multiple determines how many convolutional layers are used in the model, and it is typically set to a value between 0.33 (YOLOv5-n) and 1.33 (YOLOv5-x). For example, if the depth multiple is set to 0.33, the number of convolutional layers in the model will be roughly one-third of the default number of layers. For YOLOv5-l the model depth multiple is set to 1.0. The width multiple determines the width of the model, which is proportional to the number of filters in the convolutional layers. Increasing the width multiple results in a wider and more complex model with more parameters, while decreasing the width multiple results in a smaller and simpler model with fewer parameters. The width multiple is typically set to a value between 0.25 and 1.25, for YOLOv5-l, it is set to 1.0. In total, there are about 46.5 million parameters in YOLOv5-l.

### 2.3. Penile shaft segmentation

**Figure 4:**
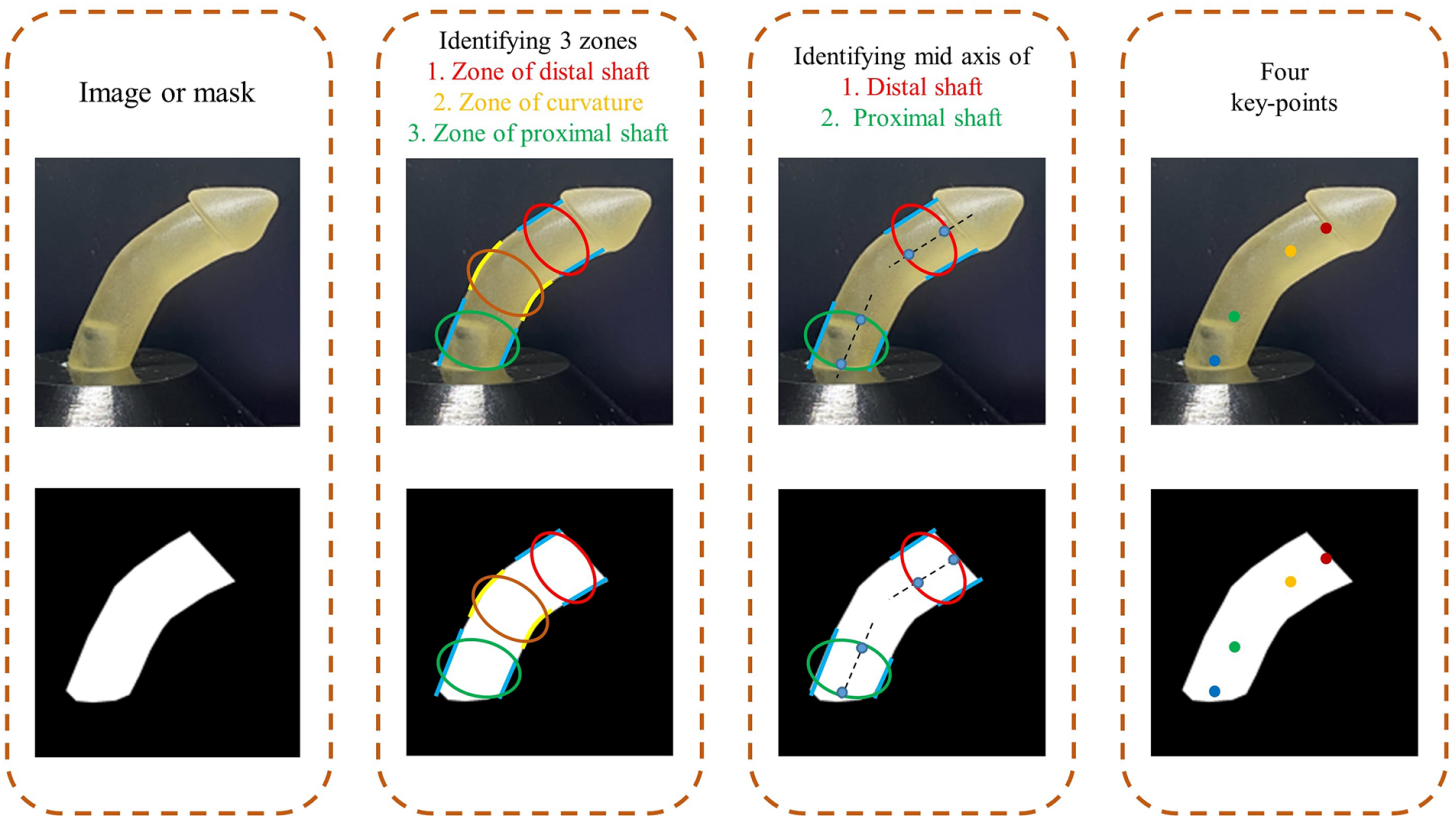
Identifying four key points of the penile shaft on cropped image and generated mask.

All cropped images were manually annotated using “labelme” (34) to mark the penile shaft (example shown in Figure 4). The images were then divided into train-test sets for the different segmentation models. We used UNet (encoder-decoder) models for the segmentation task after considering several cutting-edge designs, including UNet3+ (35), MultiResUNet (36), and Ensambled UNet (37). Different backbone networks, such as ResNet50 (38), DenseNet121 (39), inceptionv3 (40), and EfficienetNetV2M (41), were employed to assess each of these models.

In the typical U-Net design, up-sampling blocks and pooling operators are employed in the expanding decoder route and the contracting encoder path, respectively. Ensemble UNet introduces a built-in ensemble of U-Nets of varying depths in UNet++, thus enabling improved segmentation performance for varying-size objects. Additionally, in UNet 3+, each decoder layer combines smaller- and same-scale feature maps from the encoder with larger-scale feature maps from the decoder, thereby capturing both fine- and coarse-grained semantics in complete scales. To incorporate multiresolution analysis, taking inspiration from Inception family networks, MultiResUNet uses MultiRes block which replaces the convolutional layer pairs in the original U-Net. This configuration is derived from incorporating and factorizing 5 × 5 and 7 × 7 convolution operations into 3 × 3 format, then reusing these to obtain results from 3 × 3, 5 × 5 and 7 × 7 convolution operations simultaneously. Moreover, the skip connections in the UNet network may introduce some disparity between features as the encoders may offer lower-level features compared to the decoders. To overcome the semantic gap between the merged features from the encoder and decoder, convolutional layers with residual paths are employed. These are called Res paths that have 3 × 3 filters as convolution layers and 1 × 1 as the residual connection.

### 2.4. Key-points detection

To estimate penile curvature from 2D images, we tested a new technique based on identifying four key points on the penile shaft. The rationale for selecting these four points is discussed in the following section. We designated four key points for all images and then used these annotations to train and validate an HRNet deep learning model.

#### 2.4.1. Defining key-points and annotation

The penile shaft was divided into 3 three distinct zones: distal shaft, curvature region, and proximal shaft. The curvature zone is defined as bounded by two curved ventral and dorsal borders, while both the distal shaft and proximal shaft have borders defined by ventral and dorsal straight lines. To measure inclination, mid-axes were drawn through the distal and proximal shaft zones. The border points of these lines were then marked as two pairs of key points. The full process is outlined in Figure 4. This approach was used to annotate all the input images with the relevant key points (at the same time, the inclination angle of the annotations was verified to ensure this didn't deviate more than 5^°^ from ground truth).

**Figure 5:**
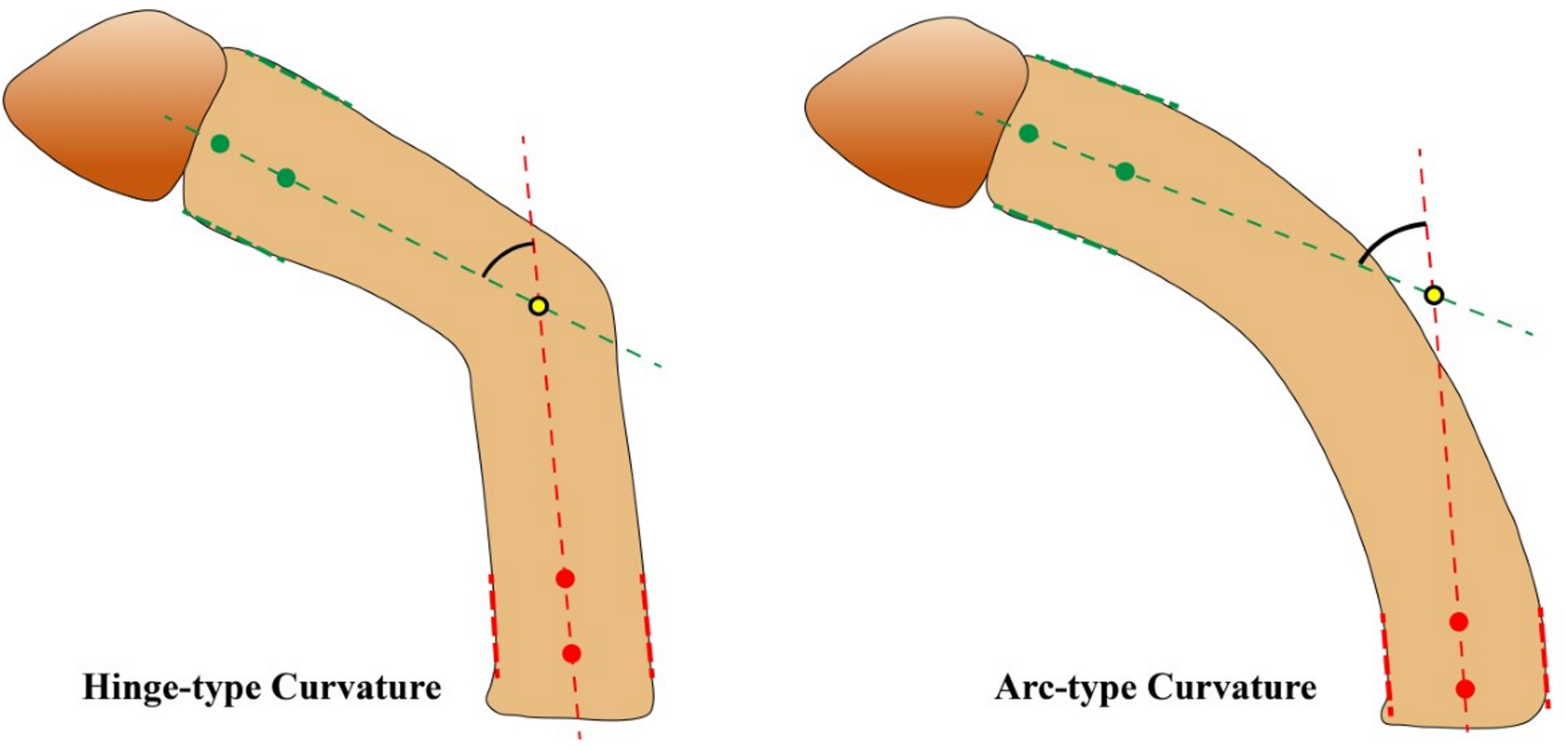
Advantage of the 4 key-points approach for hinge-type and arc-type penile shafts.

The motivation behind using the 4 dots approach instead of using the typical 3-dot one is to come up with a generalized approach for both hinge-type and arc-type penile shafts. As shown in Figure 5, defining 4 key points works for hinge-type shafts and is applicable for arc-type shafts. On the other hand, even though defining 3 points, used in previous studies (11, 31, 32, 42, 43), to measure the curvature angle could work for hinge-type shafts, it should fail in case of arc-type curvature providing misleading values.

#### 2.4.2. HRNet model

Penile shaft assessment was performed using the HRNetV2-18 CNN architecture which is designed for landmark detection (44). HRNetV2-18 is a variant of the HRNet architecture which has already been used in a variety of computer vision tasks. HRNetV2 is based on the idea of using multiple parallel “branches” of convolutional layers, each of which processes the input image at a different resolution. These branches are then combined in a “fusion” step, whereby the output of each branch is concatenated and processed by additional convolutional layers to produce the final output. This allows the network to learn features at multiple scales, which is crucial for accurate key point detection since penile shafts can vary significantly in size and appearance.

**Figure 6:**
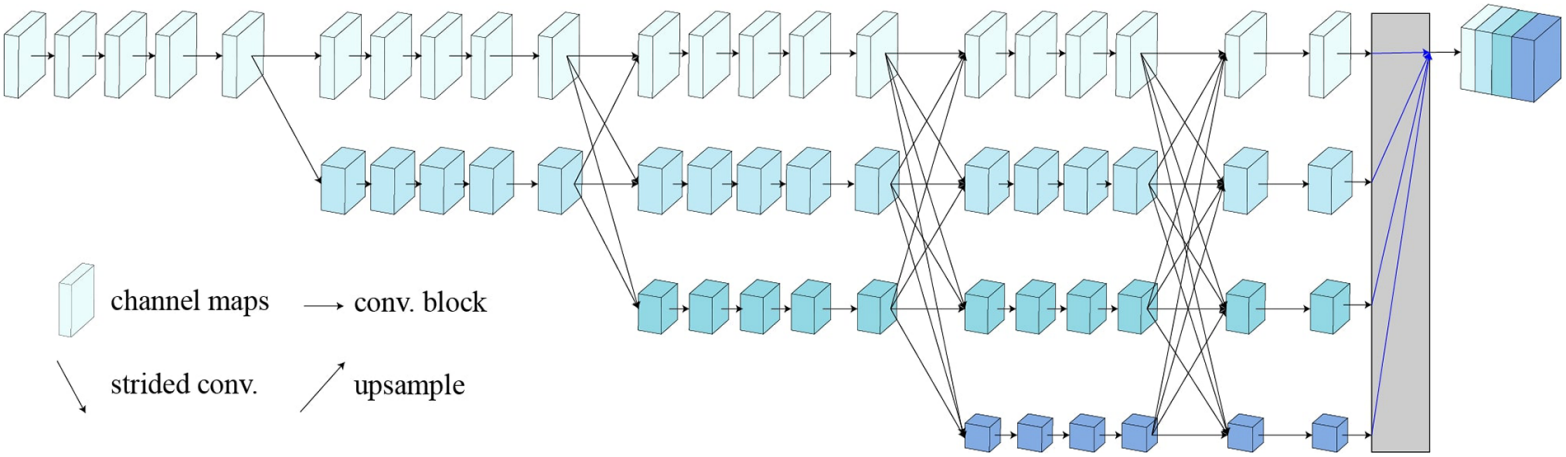
HRNetV2 architecture.

Input to the HRNetV2-18 network is first processed *via* a series of convolutional layers which reduce the spatial resolution of each image and extract low-level features. The output of these initial layers is then fed into parallel branches, where the features are further refined at different scales. Finally, outputs from the branches are concatenated and processed using additional convolutional layers to produce the final output as shown in Figure 6. Low-resolution representations are rescaled *via* bilinear up-sampling to achieve high resolution. Subsets of representations are then concatenated, resulting in high-resolution composites that can be used to estimate segmentation maps/landmark heat maps. Output representations from all four resolutions are mixed through 1 × 1 convolution to produce a final 15C-dimensional representation. For each position, the mixed representation is passed to a linear regressor with mean square error (MSE) loss to predict segmentation key-point heat maps. HRNetV2-W18 has previously been shown to achieve state-of-the-art performance in a variety of landmark detection tasks, and can accurately localize a wide range of landmarks even in very challenging scenarios (such as low-resolution images or pictures with large pose variations).

### 2.5. Angle estimation

**Figure 7:**
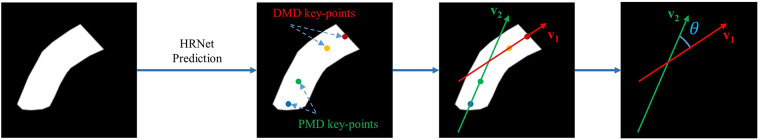
Angle calculation process using 4 predicted key points (distal mid-axis dots, DMD; and proximal mid-axis dots, PMD).

Once the HRNet model has predicted 4 key points denoting the distal mid-axis dots (DMD) and proximal mid-axis dots (PMD), we then proceed to calculate two vectors to identify the lines shown in Figure 7.

HRNet returns 4 key points: DMD_top (*x*_1_, *y*_1_), DMD_top (*x_2_*, *y*_2_), PMD_top (*x*_3_, *y*_3_) and PMD_top (*x*_4_, *y*_4_). These landmarks can then be used to calculate the distal mid-axis vector *v*_1_ and proximal mid-axis vector *v*_2_ using the following equations;

$$v_{1}=\;(x_{1}-x_{2})i+(y_{1}-y_{2})j$$

$$v_{2}=\;(x_{3}-x_{4})i+(y_{3}-y_{4})j$$

After calculating the vectors, we determined the angle between these vectors using the equation below;

$$\theta =\;\mathrm{co}s^{-1}\left(\frac{v_{1}\cdot v_{2}}{\left|v_{1}||v_{2}\right|}\right)$$

Each vector was defined by two values in an array, depicting the components on both horizontal and vertical axes, then Python was used to perform all subsequent calculations. Instead of typical slope-based angle calculation, this vector-based approach yields more reliable results by providing directional information to avoid confusion when angles approach 90°.

### 2.6. Experimental setup

For all experiments, the penile area localization and segmentation steps were performed using 5-fold cross-validation. As there were a total of 9 plastic model images, for each of the first 4 folds we had images from two plastic models and for the last fold we had images from one plastic model. For the model training, each time one fold was used as testing data and others as training data. We further split the training data in a random stratified manner keeping 20% for validation and the rest for training. For key point detection, we performed 9-fold cross-validation (7 model image sets were used for training, 1 for validation, and 1 for testing repeating 9 times). In all cases, to increase the variety of the training dataset, we randomly applied various augmentations including random horizontal flip, random brightness contrast, random gamma, random RGB shift, shift-scale-rotate, perspective shift, and rotation, thereby increasing the total number of training images 5-fold.

The YOLOv5 model used included 36 layers with 46,138,294 parameters. SGD (Stochastic gradient descent) optimizer was used with a learning rate of 0.01. A total of 100 epochs were trained with batch size 16. For detection, we used predictions with a greater than 0.75 confidence score to prevent false or multiple detections.

For the segmentation step, each model was trained in two separate phases. In the first phase, each UNet (encoder-decoder) was trained for 200 epochs while the encoder part was untrained using imagenet (45) pre-trained weights only. A model width of 16 and a model depth of 5 were used for all settings. The learning rate was 0.0001 and there was patience of 20 epochs (meaning that training will stop if the validation error doesn't decrease for 20 consecutive epochs). In the second phase, the entire model was trained for 100 epochs, unfreezing the encoder step with a low learning rate of 0.00005. Patience was set to 10 epochs and the batch size was 4. Binary Cross Entropy was used as a loss function. The best model was selected based on validation mean squared error.

In the case of HRNet training, we used 30 epochs with imagenet pre-trained weights and a batch size of 16. The optimizer used was “Adam” and the learning rate was 0.0001.

### 2.7. Testing on real images

The HRNet model was initially trained on masks from penile model images and then assessed for performance with real patient cases (using 4 intraoperative lateral penile images captured under erection test, from publicly available sources). Images were segmented manually to generate masks and the HRNet model was used to predict key points on the masks.

### 2.8. Evaluation metrics

#### 2.8.1. Object detection evaluation metric

The performance of the penile area localization network was assessed using mean average precision (mAP). AP is the area under the precision-recall curve, and mAP is the average AP across all classes. mAP@0.5 indicates that the average AP for IoU (Intersection over Union) is 0.5, while mAP@[.5:.95] corresponds to the average AP for IoU from 0.5 to 0.95, with a step size of 0.05.

$$\mathrm{mAP}=\;\frac{1}{n}\sum_{i=1}^{n}AP_{i}$$

where *n* is the number of classes (in this case only one: the penile area).

#### 2.8.2. Segmentation evaluation metrics

Three assessment metrics—model accuracy, intersection over union, and dice similarity coefficient—were used to assess the performance of the shaft segmentation networks. The definitions of these performance measures are given below.

$$\mathrm{DSC}=\;\frac{2\mathrm{TP}}{2\mathrm{TP}+\mathrm{FP}+\mathrm{FN}}$$

The counts of true positive (TP), false positive (FP), true negative (TN), and false negative (FN) pixels.

$$\mathrm{IoU}=\;\frac{\mathrm{TP}}{\mathrm{TP}+\mathrm{FP}+\mathrm{FN}}$$

$$\mathrm{Accuracy}=\;\frac{\mathrm{TP}+\mathrm{TN}}{\mathrm{TP}+\mathrm{TN}+\mathrm{FP}+\mathrm{FN}}$$

It should be noted that both IoU and DSC provide a quantitative assessment of the overlap between the segmentation masks used for prediction and those used for ground truth, with the main difference being that DSC gives true shaft prediction pixels a 2-fold advantage over IoU. For this study, we calculated weighted IoU since both the mask and background had almost equal distribution in the cropped 256 × 256 pixel images. All three assessment metrics were evaluated on a per-image basis. Accuracy, IoU, and DSC were calculated for each mask generated.

#### 2.8.3. Key point detection

NME (Normalized Mean Error) was the primary evaluation criterion for key point designation on the penile shaft. This measure calculates the Euclidean distance between ground truth points and the predicted points, then divides this distance by a normalized factor. The formula is as follows:

$$\mathrm{NME}(P,\hat{P})=\;\frac{1}{N}\sum_{i=1}^{N}\frac{{\left|\left|\;p_{i}-\hat{\;p_{i}}\right|\right|}_{2}}{d}$$

where *P* and $\hat{P}$ denote the predicted and ground-truth coordinates of key points, respectively. N is the number of points, and d is the reference distance to normalize the absolute errors. In this case, the reference distance was taken from the top DMD point to the bottom PMD point.

#### 2.8.4. Curvature angle estimation evaluation metrics

The primary scoring system used for curvature angle estimation was a mean absolute error (MAE) and is defined by:

$$\mathrm{MAE}=\;\frac{1}{n}\sum_{i=1}^{n}\left|{\tilde{y}}_{i}-y_{i}\right|$$

where *n* is the total number of examples, ${\tilde{y}}_{i}$ is the estimated curvature angle averaged over all predictions for one penile model, and $y_{i}$ is the ground truth value for that same model. Individual error values were calculated for each image and then divided by the total number of images to obtain the overall MAE.

## 3. Results

Shaft segmentation networks, curvature estimation technique, and penile localization model were thoroughly evaluated both numerically and qualitatively as part of the AI framework's performance testing.

### 3.1. Penile area localization

YOLOv5l performed very well in detecting the penile area with an average mAP0.5 of 99.4% for 5 folds, and a mAP0.5–0.95 value of 73.8%. The fold-wise results are given in Table 1 and indicate that the model did not fail in the assessment of any input image (although small differences in bounding boxes may have caused minor fluctuations in mAP). Other than fold_1, for all cases, the model almost perfectly predicted bounding boxes with 50% overlap in IoU.

**Table 1:** YOLOv5l prediction mAP (mean average precision) for each fold.

Fold	mAP0.5	mAP50-95
fold_0	0.995	0.693
fold_1	0.991	0.692
fold_2	0.995	0.777
fold_3	0.995	0.713
fold_4	0.995	0.815
Avg.	**0** **.** **994**	**0** **.** **738**

### 3.2. Shaft segmentation

Table 2 provides the segmentation results for all test cases when using UNetE, UNet3P, and MultiResUNet as decoders, and DenseNet121, ResNet50, InceptionV3 and EfficientNetV2M as encoders. For all the encoder and decoder combinations models were trained and test scores were determined. Among all models, the combination of Ensambled UNet (UNetE) and DenseNet121 performed the best, with an average IoU of 96.43% for 5 folds. The DSC score and the accuracy were also superior to other models, scoring 94.50% and 98.12% respectively. In comparison to encoders based on other designs, DenseNet encoders displayed greater levels of performance. This may be due to the broad interconnectedness afforded by thick layers as well as the collective knowledge provided by preceding layers. The use of an ensemble U-Net model architecture may also have improved the performance of the segmentation network by increasing capacity, improving generalization, reducing overfitting, and increasing robustness. By training multiple U-Net models on different subsets of data, and then averaging the predictions obtained, Ensemble U-Net could potentially achieve better generalization with unseen data. In particular, Ensemble U-Net could reduce overfitting by averaging the predictions of multiple models, as well as being more resistant to noise and other variations in the input data (again due to averaging out these effects across multiple models).

**Table 2:** Segmentation results including IoU (intersection over union) and DSC (dice similarity coefficient).

Segmentation model	Encoder	Accuracy	IoU	DSC
UNet3P	DenseNet121	97.88 ± 0.32	96.01 ± 0.60	93.84 ± 0.93
	ResNet50	96.70 ± 1.78	93.99 ± 2.92	90.97 ± 3.43
	EfficentNetV2M	97.35 ± 0.87	95.09 ± 1.49	92.53 ± 1.69
	InceptionV3	97.84 ± 0.30	95.94 ± 0.56	93.74 ± 0.86
MultiResUnet	DenseNet121	97.99 ± 0.31	96.20 ± 0.56	94.14 ± 0.80
	ResNet50	97.86 ± 0.57	95.97 ± 0.99	93.79 ± 1.24
	EfficentNetV2M	97.58 ± 0.91	95.48 ± 1.52	93.08 ± 1.95
	InceptionV3	98.01 ± 0.26	96.24 ± 0.47	94.19 ± 0.82
UNetE	DenseNet121	**98.12 ** **±** ** 0.31**	**96.43 ** **±** ** 0.57**	**94.50 ** **±** ** 0.75**
	ResNet50	97.84 ± 0.52	95.93 ± 0.92	93.74 ± 1.18
	EfficentNetV2M	97.53 ± 0.62	95.40 ± 1.07	92.94 ± 1.34
	InceptionV3	97.98 ± 0.33	96.18 ± 0.61	94.11 ± 0.84

### 3.3. Key point detection and angle estimation

HRNet performed very well in the detection of key points on both penile model images and segmentation masks. The average test NME (Normalized Mean Error) between ground truth key points and predicted key points was 0.0708 for the images and 0.0430 for derivative masks. For each fold, the predicted angles for individual model images were determined and the results are shown in Table 3. Overall MAE for the angles predicted from penile model images was approximately 4.5°.

**Table 3:** Angle prediction results and MAE (mean average error) for penile model images.

Test case	Ground Truth	Predicted Angle	MAE
pModel_1	75	75.63684±3.908193	3.031769
pModel_2	33	32.88968±3.08491	2.312334
pModel_3	82	77.54307 ±9.729288	6.782767
pModel_4	40	43.14194 ±3.134882	3.503608
pModel_5	58	57.01167 ±4.751162	3.992647
pModel_6	50	48.53089 ±3.796258	3.372716
pModel_7	86	82.17514±7.00086	6.12593
pModel_8	60	64.60267 ±8.151951	7.484112
pModel_9	18	14.52068 ±3.524402	3.925218
		**Overall:**	**4** **.** **522118**

**Figure 8:**
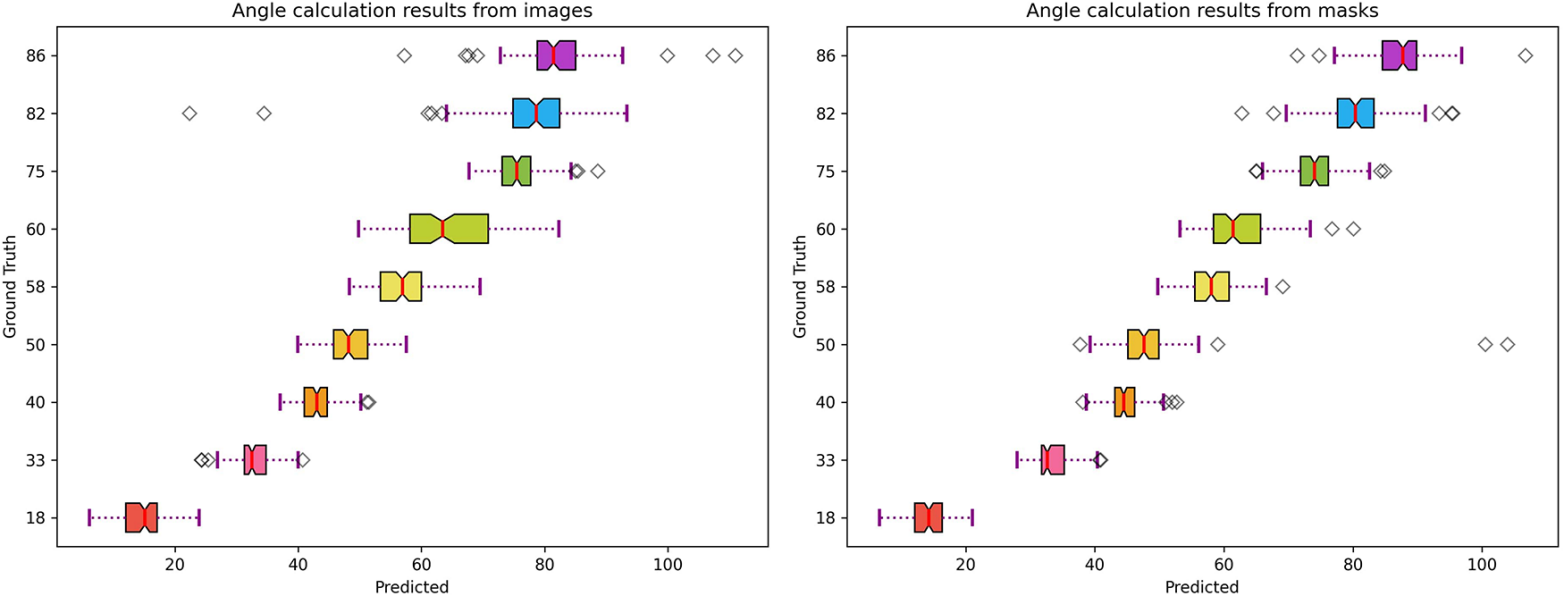
Curvature angle estimation performance from penile model images and derivative masks.

Angle predictions from segmentation masks were superior to those obtained from penile model images, with an overall MAE of just 3.8° as shown in Table 4. Figure 8 displays the improvement in predictions achieved when using masks instead of original images (as indicated by lower standard deviation and consistent angle prediction for all images from the same model). Overall, model performance outperforms the previous study of penile angle calculation using the same dataset by Abbas et al. (32). While that study showed an overall MAE of 8.53, we could achieve as less as 3.81 which is almost 2.24 times better. Compared with previous studies of penile curvature using plastic models, the novel pipeline reported here was also more accurate than Goniometer and/or UVI approaches where the mean error was up to 13.6 (17).

**Table 4:** Angle prediction results and MAE (mean average error) for segmentation masks.

Test case	Ground Truth	Predicted Angle	MAE
pModel_1	75	74.15353 ± 3.895762	3.041326
pModel_2	33	33.47152 ± 2.812504	2.156159
pModel_3	82	80.39 399 ±5.277415	4.156524
pModel_4	40	44.77705 ± 2.881157	4.849213
pModel_5	58	58.15946 ± 3.736759	3.004913
pModel_6	50	48.60104 ± 8.772863	4.758214
pModel_7	86	87.01886 ± 4.666506	3.596134
pModel_8	60	62.63402 ± 5.544223	4.609684
pModel_9	18	14.08197 ± 3.026902	4.163569
		**Overall:**	**3** **.** **813667**

**Figure 9:**
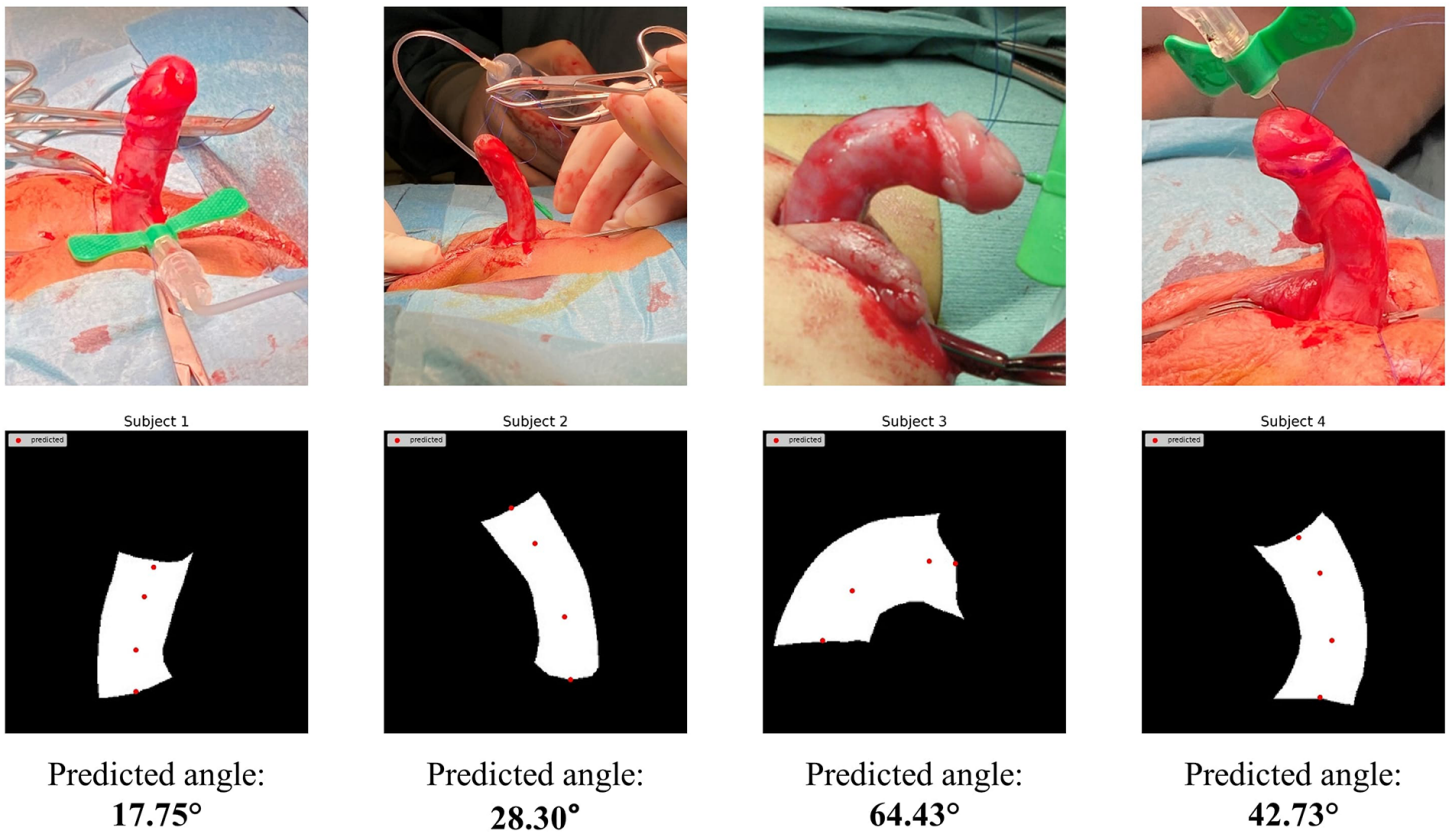
HRNet key-point detection results using masks manually extracted from real patient images.

Finally, we proceeded to test model performance using real patient masks as shown in Figure 9. Despite having been trained on masks from penile models, our HRNet-based tool was able to successfully predict both DMD and PMD landmarks on the real patient masks. The angle calculations generated from these masks were also comparable with manual image assessment by a clinical expert using the mobile application Angle 360 (Table 5).

**Table 5:** Comparison between human expert vs. AI for penile angle prediction.

Subject	Prediction by HRNet	Prediction by a medical expert
1	17.75°	23.82°
2	28.30°	29.99°
3	64.43°	69.79°
4	42.73°	44.93°

## 4. Discussion

Rapid advances in computational power have ensured that AI is gaining ever more popularity for the automation of routine clinical tasks. AI now offers the opportunity to build highly accurate models that enable precise and timely examination of medical images. Abbas et al*.* (32), previously aimed to automate curvature estimations from 2D images based on localization and segmentation of the penile shaft, but subsequent angle calculation displayed several limitations. In the current study, rather than use a hard-coded approach, we instead developed a novel deep learning-based algorithm that can robustly calculate the extent of penile curvature with a high level of accuracy.

Penile curvature (PC) assessment is not standardized and remains prone to considerable variability and subjectivity (46). Typical measurement processes are UVI (Unaided Visual Inspection) or by Goniometer, which have proven distinctly unreliable. In a previous study by Villanueva et al*.*, the mean errors for all PC measurement techniques ranged from 3.5° to 13.6°, with no significant difference between UVI and goniometry procedures (17). Since surgeons cannot reliably evaluate PC, and there are currently no guidelines for real-time intraoperative measurement of curvature, there is a clear unmet clinical need to develop more robust methods of assessing PC. Accordingly, Fernandez et al*.* (43), attempted to standardize curvature measurement from 2D images in a semi-automated manner, but the resultant algorithm depended on identifying the geometric centre of the penile shaft, which can vary significantly from patient to patient. In addition, this process still required direct human intervention, hence results could vary markedly depending on user expertise. Similarly, Villanueva et al*.* (14), used an app-based approach to calculate curvature angles from 2D images, but again the same technical limitations prevent wider application of this method.

In previous work, Abbas et al*.* (32), proposed a fully automated, end-to-end application that could predict PC extent from captured images, but the hard-coded angle calculation step was unreliable when applied to real-life cases (which displays highly variable shaft size and shape, unlike the uniform plastic models used in initial testing). Additionally, the slope-based calculation was found to give erroneous results when angles approached 90° (since the tangent value of 90° is undefined). To overcome the limitations of previous studies, here we developed a new algorithm in which angle calculation no longer depends on identifying the curved region or centre point of “maximum” curvature. Using deep learning models instead of typical image analysis approaches, we achieved substantial improvement in angle predictions and then proceeded to test performance using shaft masks from real patients. The deep learning process showed moderate accuracy, indicating potentiality for translation into real-life scenarios. To achieve this goal, further model development will require: (1) a large dataset of penile curvature images from real-life patients, and/or (2) an improved segmentation step that can predict shaft masks with similar accuracy in both plastic models and real patients. A few limitations of this study should also be noted. In particular, camera angle and picture quality can impact mask generation and angle calculation, although this process should perform well for images taken from a lateral view under well-lit conditions. Also, segmentation of real-life anatomy is more challenging due to excess dartos, soft tissues, blood etc. Despite these drawbacks, this study successfully developed a novel and accurate framework for automated penile curvature measurement in regulated circumstances.

## 5. Conclusion

We devised an innovative AI-based approach to perform high-accuracy automatic measurements of PC. This technique uses deep neural networks to segment the penile shaft from captured images and then employs another deep learning network to determine the curvature angle. These findings are superior to those obtained *via* physical examination by urologists and can be accomplished in a far shorter amount of time. Our findings indicate that AI-based approaches may provide accurate, reliable, and generally accessible methods of measuring PC, which might address several flaws present in current assessment methods. The approach discussed in this article may not yet be ready for clinical application, but represents a significant step towards real-time automated PC monitoring in clinical settings.

## Data Availability

The datasets generated during and/or analyzed during the current study are available from the corresponding author upon reasonable request. Requests to access these datasets should be directed to Tariq O. Abbas, tariq2c@hotmail.com.
