# Supplementary material for: Automated measurement of penile curvature using deep learning-based novel quantification method
Source: Front Pediatr. 2023 Apr 17;11:1149318. doi: 10.3389/fped.2023.1149318 (PMC10150132; doi:10.3389/fped.2023.1149318)
Supplement: Supplementary file 1 [file Table1.docx]

Supplementary Material

**Precise Automation of Penile Curvature Measurement Using Deep Learning with a Novel Quantification Method**

Sriman Bidhan Baray, Mohamed AbdelMoniem, Sakib Mahmud, Saidul Kabir, Md. Ahasan Atick Faisal, Muhammad E. H. Chowdhury, Tariq O. Abbas

***Correspondence:**

Tariq O. Abbas; Urology Division, Surgery Department, Sidra Medicine, Doha, Qatar
[tariq2c@hotmail.com](mailto:tariq2c@hotmail.com)

# Supplementary Figures and Tables

Table 1 Angles and number of images for each penile model in the dataset

| **Penile model** | **Angle** | **Number of images** |
| --- | --- | --- |
| pModel_1 | 75$^{\circ}$ | 104 |
| pModel_2 | 33$^{\circ}$ | 100 |
| pModel_3 | 82$^{\circ}$ | 100 |
| pModel_4 | 40$^{\circ}$ | 102 |
| pModel_5 | 58$^{\circ}$ | 103 |
| pModel_6 | 50$^{\circ}$ | 97 |
| 1pModel_7 | 86$^{\circ}$ | 101 |
| pModel_8 | 60$^{\circ}$ | 107 |
| pModel_9 | 18$^{\circ}$ | 99 |

| 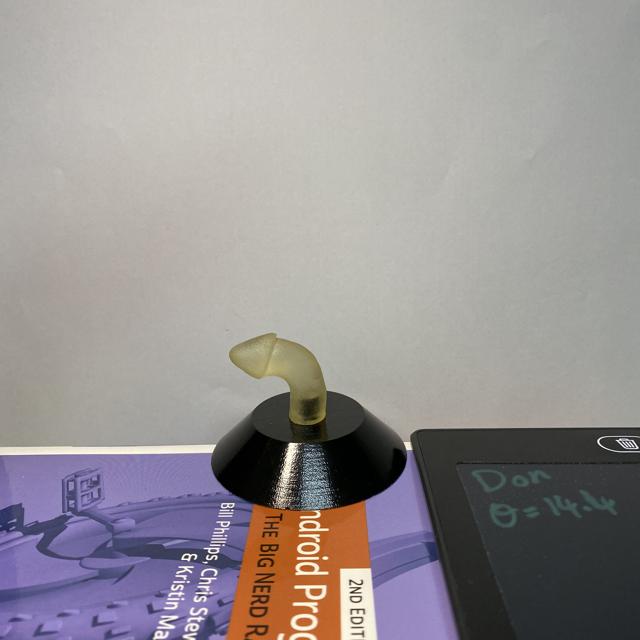  75° | 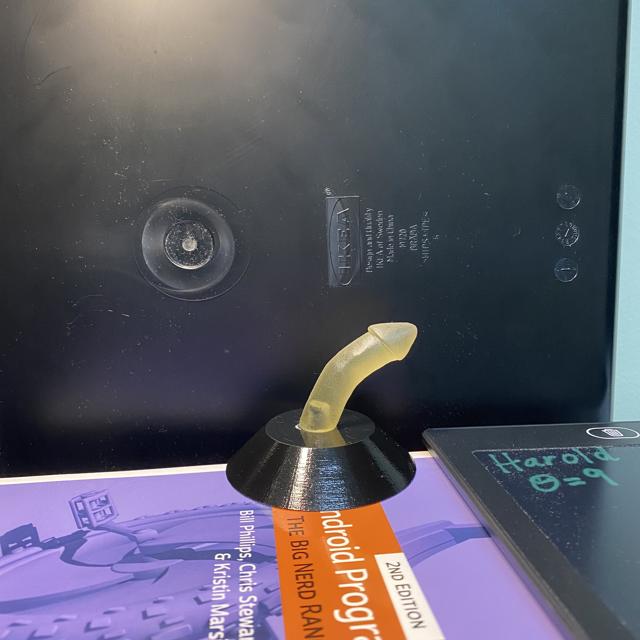  33° | 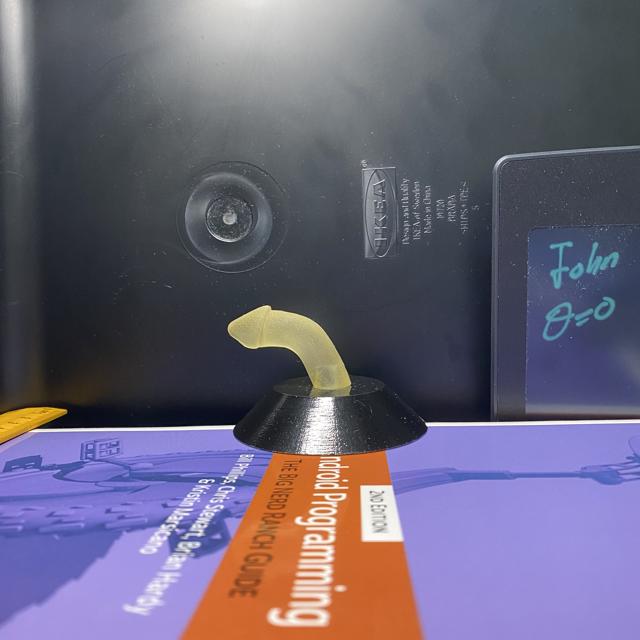  40° |
| --- | --- | --- |
| 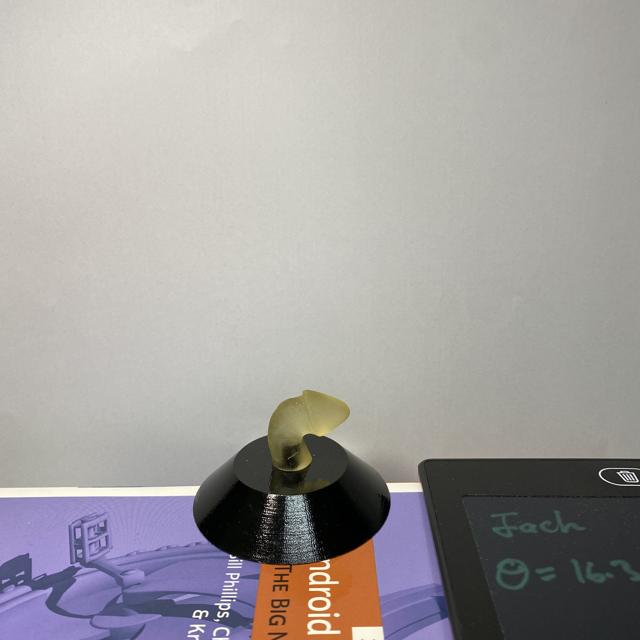  82° | 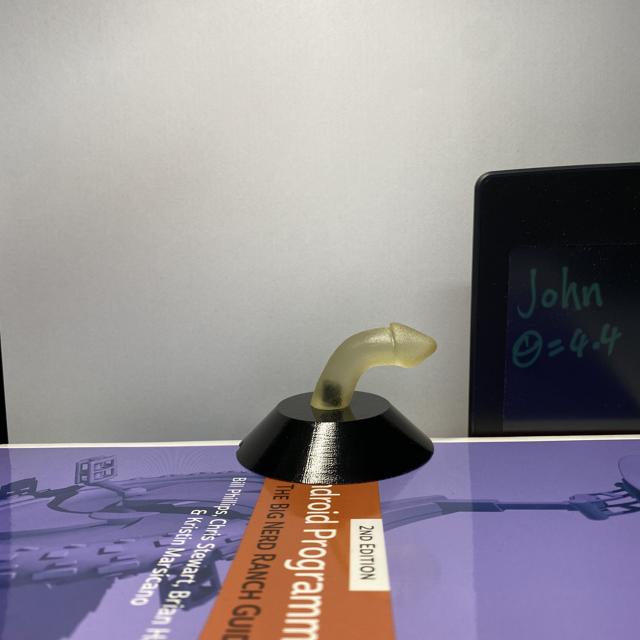  58° | 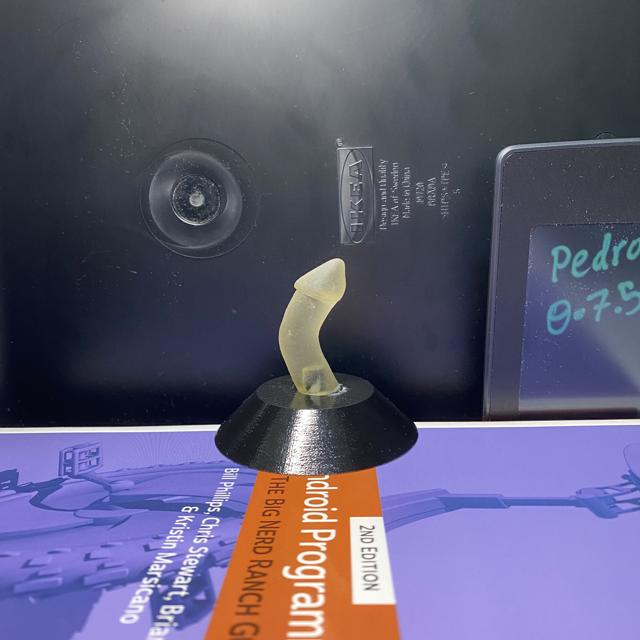  50° |
| 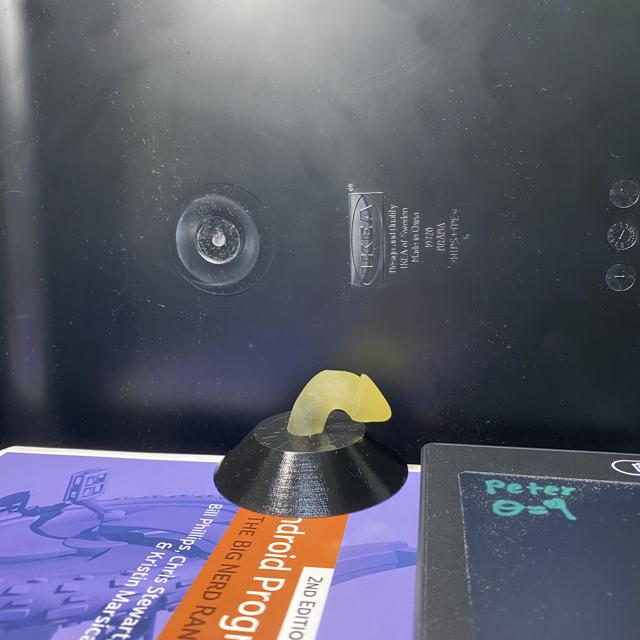  86° | 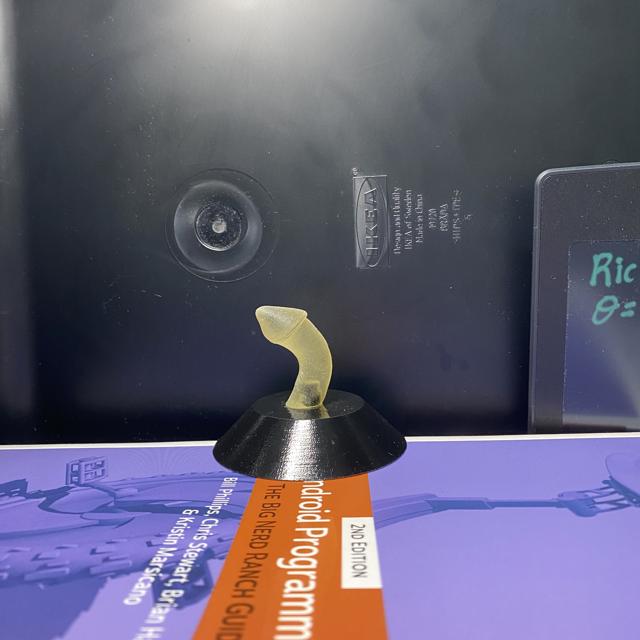  60° | 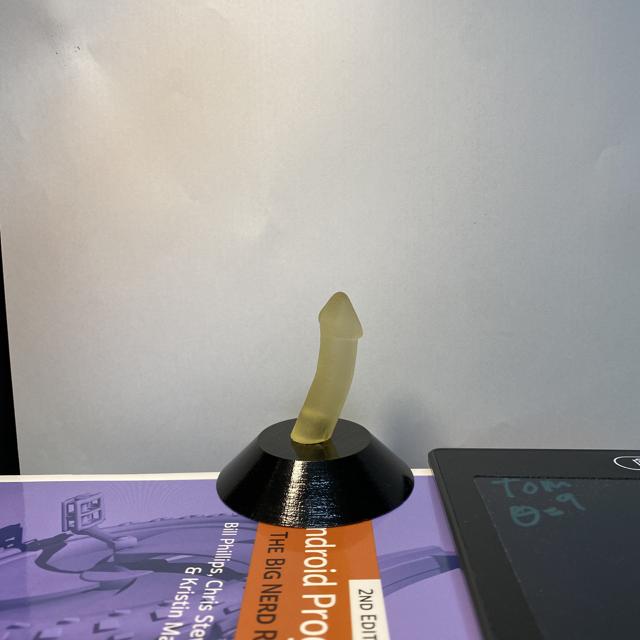  18° |

Figure 1 Sample images of each penile model present in the dataset.
